# Sarcopenia in Rheumatic Diseases: A Hidden Issue of Concern

**DOI:** 10.3390/diseases13050134

**Published:** 2025-04-26

**Authors:** Eleni C. Pardali, Markos Klonizakis, Dimitrios G. Goulis, Sousana K. Papadopoulou, Christos Cholevas, Constantinos Giaginis, Christina Tsigalou, Dimitrios P. Bogdanos, Maria G. Grammatikopoulou

**Affiliations:** 1 Unit of Immunonutrition, Department of Rheumatology and Clinical Immunology, University General Hospital of Larissa, Faculty of Medicine, School of Health Sciences, University of Thessaly, Biopolis Campus, GR-42110 Larissa, Greece; elpardali@uth.gr (E.C.P.); bogdanos@med.uth.gr (D.P.B.); 2 Lifestyle, Exercise and Nutrition Improvement (LENI) Research Group, Department of Nursing and Midwifery, Sheffield Hallam University, Collegiate Hall, Collegiate Crescent Rd, Sheffield S10 2BP, UK; m.klonizakis@shu.ac.uk; 3 Unit of Reproductive Endocrinology, 1st Department of Obstetrics and Gynecology, Faculty of Health, Sciences, Medical School, Aristotle University of Thessaloniki, GR-54124 Thessaloniki, Greece; 4 Department of Nutritional Sciences and Dietetics, Faculty of Health Sciences, International Hellenic University, GR-57400 Thessaloniki, Greece; sousana@ihu.gr; 5 First Department of Ophthalmology, Medical School, Aristotle University of Thessaloniki, AHEPA University Hospital, University Campus, GR-54636 Thessaloniki, Greece; ccholevas@auth.gr; 6 Department of Food Science and Nutrition, School of the Environment, University of the Aegean, GR-81400 Lemnos, Greece; cgiaginis@aegean.gr; 7 Laboratory of Hygiene and Environmental Protection, Medical School, Democritus University of Thrace, University Hospital, GR-68100 Alexandroupolis, Greece; xtsigalou@yahoo.gr

**Keywords:** rheumatic diseases, juvenile arthritis, scleroderma, dietary supplements, systemic lupus erythematosus, cachexia, dynapenia, psoriatic arthritis

## Abstract

Sarcopenia is characterized by a loss of muscle mass and function, with significant implications for the physical performance of the affected people. Although commonly associated with aging, disease-related sarcopenia is of great clinical importance, particularly as it impacts disease progression and outcomes. Individuals with rheumatic diseases (RDs), including rheumatoid arthritis, systemic sclerosis, spondyloarthritides, systemic lupus erythematosus, fibromyalgia, myositis, or vasculitis, exhibit a high prevalence of sarcopenia, which exacerbates their clinical symptoms and contributes to poorer disease outcomes. Chronic inflammation influences muscle tissue degradation, causing a decline in physical performance. Apart from the apparent clinical manifestations, patients with RDs also use pharmacological treatments that negatively impact muscle mass further, increasing the risk of sarcopenia. Nutrition (diet and dietary supplements) and exercise interventions have been recommended as protective measures for sarcopenia as they may mitigate its adverse events. The present narrative review seeks to explore the methods used to assess sarcopenia in patients with RDs, its prevalence among them, and the challenges faced by the affected individuals, while critically assessing the appropriateness and limitations of current sarcopenia assessment tools in the context of RDs.

## 1. Introduction

Sarcopenia (Greek ‘sarx’ or flesh + ‘penia’ or loss) was first proposed by Irwin Rosenberg in 1989 as a term describing age-related muscle loss [1,2]. In 2010, the European Working Group on Sarcopenia in Older People (EWGSOP) defined sarcopenia as a musculoskeletal disorder characterized by muscle mass loss and reduced muscle function, mostly affecting older adults [3]. In 2019, the EWGSOP criteria were updated, widening the age range of patients affected by sarcopenia [4]. The revised definition emphasized reduced muscle strength as a primary focus for identifying sarcopenia, with a diagnosis being confirmed when low muscle mass or impaired physical performance are present [4]. Sarcopenia is broadly classified into two types: primary, which is age-related, and secondary, which is disease-related [3].

Older adults present an increased prevalence of sarcopenia, leading to frailty and disability [5], as well as greater mortality rates [6]. All these parameters contribute to an increased incidence of hospitalizations and a high economic burden, highlighting the impact of sarcopenia on the healthcare and societal systems [7].

**Figure 1:**
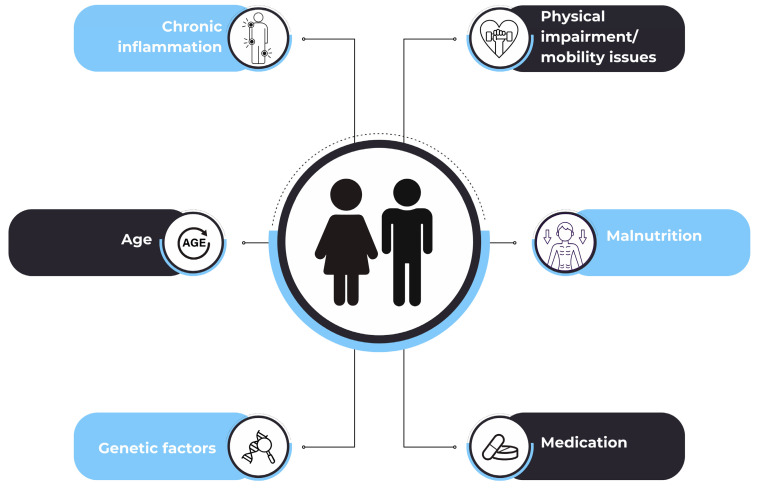
Factors contributing to sarcopenia in rheumatic diseases.

Rheumatic diseases (RDs) are characterized by increased inflammation that mostly affects joints, bones, and cartilage, leading to pain, dysmotility, physical dysfunction, and compromised well-being [8]. Chronic inflammation and impaired physical capability are also associated with the development of sarcopenia [3]. When patients with RDs present myopenia [9,10] and cachexia [11], they are at greater risk of developing sarcopenia (Figure 1), which, in turn, may increase adverse events and lead to poorer disease outcomes. Genetic predisposition plays an important role in modulating immune-inflammatory pathways that connect joint inflammation with the muscle deterioration observed in sarcopenia [12]. These inflammatory processes can trigger epigenetic changes and disrupt gene expression, ultimately contributing to muscle wasting, degradation, and frailty [13]. Pro-inflammatory cytokines, such as interleukin-6 (IL-6) and tumor-necrosis factor-α (TNF-α), disrupt muscle homeostasis, leading to muscle deterioration [14]. Drug-induced sarcopenia represents a form of secondary sarcopenia [15], as several therapeutic agents —including glucocorticoids (GCs), commonly used in the treatment of RDs [16]— have been shown to adversely affect muscle metabolism and contribute to muscle wasting through various catabolic pathways [17,18].

All the aforementioned factors—combined with the reduced quality of life commonly observed in patients with RDs [19]—place this population at increased risk of developing sarcopenia. Furthermore, there are currently no universally accepted diagnostic criteria for sarcopenia, and existing methods may not be fully applicable or reliable in this context. For instance, handgrip strength (HGS), a commonly used measure of muscle function, may be impractical or inaccurate in patients with rheumatoid arthritis (RA) due to joint deformities and impaired hand function. Therefore, it is crucial to assess the prevalence of sarcopenia in individuals with RDs and evaluate the suitability and validity of existing diagnostic criteria within this specific population.

The present narrative review seeks to compile all published evidence regarding the prevalence and importance of sarcopenia in RDs, primarily in patient-centered health outcomes and its broader economic impact on society.

## 2. Literature Search

A comprehensive search was performed on PubMed up to February 2025, for all available primary research on sarcopenia in patients with RDs.

## 3. Sarcopenia Definitions

Several scientific groups have made efforts to define sarcopenia and suggest diagnostic criteria [20]. The first organized effort was conducted in the year 2010 by the Special Interest Group of the European Society for Clinical Nutrition and Metabolism (ESPEN) [21]. Sarcopenia was defined as a loss of muscle mass and strength, in alignment to Rosenberg’s characterization. Nonetheless, this definition lacked the proposition of a specific tool to assess muscle mass, resulting in frequent misdiagnosis of the condition.

In the same year, the EWGSOP published their consensus definition in line with the ESPEN. It pioneered the use of muscle function and specific threshold values to improve the precision and reliability of sarcopenia diagnosis [3].

The International Working Group on Sarcopenia also defined sarcopenia as an age-related condition characterized by low muscle mass and physical impairment [22]. This definition lacks the inclusion of muscle strength, a key and sensitive parameter in the assessment of sarcopenia, and employs higher threshold values, a fact that may increase the likelihood of overdiagnosis.

On the other hand, the Society of Sarcopenia, Cachexia and Wasting, to provide a universally acceptable definition, defined sarcopenia as low muscle mass and limited mobility [23]. Likewise, this definition does not account for the effect of the condition on muscle strength. Still, it may have higher specificity given that muscle mass thresholds are adjusted to ethnic and age groups.

The Asian Working Group for Sarcopenia (AWGS) proposed a diagnostic algorithm tailored to Asian populations due to the anthropometric differences observed between ethnicities [24]. While similar to the criteria for age-related sarcopenia, it incorporated region-specific data, established clear threshold values for diagnostic components, and refined measurement protocols, allowing for a more accurate application in this population, while expanding sarcopenia screening in several clinical conditions [24].

More stringent diagnostic criteria were proposed by the Foundation for the National Institutes of Health Sarcopenia Project (FNIH), derived from large and diverse cohorts, which appeared to decrease the number of patients screening positive [25].

In the year 2019, the EWGSOP updated its criteria, emphasizing muscle strength as a key indicator and expanding sarcopenia beyond age-related conditions to include anyone with low muscle strength [4].

Following the EWGSOP, the AWGS revised its diagnostic algorithm, introducing the term “possible sarcopenia” and using the SARC-F tool [26]. The new criteria introduced stricter thresholds and more precise assessment methods while providing alternatives to assess sarcopenia under various circumstances. The classification of sarcopenia into distinct categories facilitates prompt diagnosis and enables personalized treatment, based on the individual patient’s needs.

The most recent definition of the condition was proposed by the Sarcopenia Definitions and Outcomes Consortium, prioritizing physical performance over muscle mass assessment, measured by HGS and walking speed [27]. This approach enhanced the precision of diagnosis, particularly for secondary sarcopenia, where mobility and function may not align with muscle mass. The various existing definitions contribute to significant variability in prevalence, based on the applied criteria.

**Figure 2:**
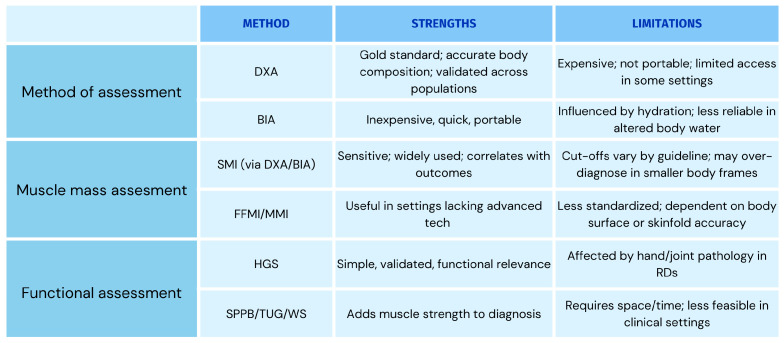
Insights and future directions for assessing sarcopenia in rheumatic diseases. BIA: bioelectrical impedance analysis; DXA: dual-energy X-ray absorptiometry; FFMI: fat-free mass index; HGS: hand grip strength; MMI: muscle mass index; RD: rheumatic disease; SMI: skeletal muscle index; SPPB: short physical performance battery; TUG: timed up and go; WS: walking speed.

The diagnostic criteria employed for assessing sarcopenia are summarized in Table 1. Figure 2 offers insights and highlights future directions for assessing sarcopenia in RDs.

## 4. Inflammation Biomarkers in Sarcopenia

Pro-inflammatory cytokines are involved in muscle wasting through several mechanisms and cascades of reactions [28]. They promote protein breakdown, disrupting the balance of muscle synthesis, leading to muscle loss [29]. Both IL-6 and TNF-α are linked to reduced muscle mass and impaired muscle strength [14]. In parallel, TNF-α triggers the activation of nuclear factor-kappa B (NF-κB), which initiates a chain of events leading to muscle cell apoptosis [30], by downregulating myosynthetic enzymes like *MyoD* and *Myogenin* [31] and upregulating the myolytic ones, including *Atrogin1* and *MuRF1* [32]. IL-6 suppresses the insulin-like growth factor 1 (IGF-1), activating the Janus kinase (JAK)–signal transducer pathway and increasing myolytic enzyme expression. The latter leads to muscle wasting and impaired muscle regeneration [33]. On the other hand, IL-1β triggers catabolic effects in muscle tissue, as it can bind to its receptors in the sarcolemma, further contributing to muscle wasting [34].

Acute-phase C-reactive protein (CRP) is also observed in elevated concentrations in people with increased muscle degradation [35]. Additionally, higher serum CRP concentrations have been linked to sarcopenia and frailty [36]. Elevated interleukin-8 (IL-8) levels, which are associated with inflammation and innate immune system activity [37], have also been shown to promote neutrophil dysregulation, leading to tissue damage. When such damage is located within the muscle tissue, it results in muscle degradation and the development of sarcopenia [38].

As for adipokines, a reduced adiponectin-to-leptin ratio has also been proposed as a potential marker of muscle damage and aging [38]. Adiponectin exerts its anti-inflammatory role by inhibiting NF-kB. At the same time, leptin—a pro-inflammatory adipokine released by the fat mass—promotes the secretion of IL-6 and TNF-α, contributing to the activation of natural killer lymphocytes [39]. An increased neutrophil-to-lymphocyte ratio has also been observed in individuals with a high risk of developing sarcopenia, which was inversely associated with fat-free mass [40]. Elevated erythrocyte sedimentation rate (ESR) has also been related to sarcopenia, reduced muscle strength, and low physical performance [41]. Table 2 provides a summary of the inflammation-related biomarkers associated with RDs, implicated in the pathophysiology of sarcopenia.

## 5. Sarcopenia and RDs

### 5.1. Sarcopenia in RA

RA is one of the most common autoimmune diseases and affects the small, symmetric diarthrodial joints in the upper and lower extremities, often accompanied by clinical manifestations and comorbidities that impact the overall quality of life of patients [42]. Patients with RA often experience myopenia [10], malnutrition [43], and cachexia [11], conditions characterized by underlying muscle loss. The latter is a defining feature of sarcopenia, ultimately contributing to its development [3].

Several studies have explored the prevalence and implications of sarcopenia in RA (Table 3), shedding light on its effects in this patient group. Women appear to be particularly affected by sarcopenia during the menopausal transition, with a high prevalence, ranging between 15.8% [44] and 62.7% [45], depending on the definition used. In RA, sarcopenia has also been associated with reduced HGS and low physical activity levels [46,47], a higher prevalence of osteoporosis [48,49], lower gait speed [50], as well as a higher inflammation status (IL-1α, IL-6, and TNF-β) [44,51]. More recent studies have associated sarcopenia in RA with poly-autoimmunity [52], body mass and body mass index (BMI), disease duration, glucocorticoid use, cumulative dose of prednisone equivalent, and lower Health Assessment Questionnaire (HAQ) scores [53,54].

Research shows that the underlying chronic inflammation in RA initiates muscle degradation, driving ectopic fat deposition [10], while myopenia is associated with increased joint damage [55], further complicating sarcopenia. Recently, Qu and associates constructed a nomogram model to predict sarcopenia in RA using BMI, disease duration, hemoglobin, and HGS [56]. This model demonstrated high accuracy and strong discriminative ability, as supported by the area under the curve and the decision curve analysis. In addition, its practical applicability is enhanced by the ease of obtaining the required clinical variables, which increases its accessibility for use in clinical settings [56].

When comparing diagnostic methods in patients with RA (Table 3), the EWGSOP 2010 criteria seem to yield a higher prevalence of sarcopenia [45,54,57,58] compared to the updated EWGSOP 2019 ones [44,45,47,53,59,60], possibly due to differences in the threshold values used. Notably, the AWGS 2014 criteria produced the greatest prevalence rates overall [48,57,61,62,63], potentially reflecting population-specific thresholds and broader diagnostic inclusion. As a matter of fact, the Asian population had previously been reported to present higher rates of sarcopenia compared to other ethnicities [64]. In contrast, the FNIH criteria demonstrated the lowest prevalence rates [53,60], consistent with its more stringent and conservative diagnostic approach.

**Table 3:** Studies assessing the prevalence of sarcopenia in patients with RA.

First Author	Sample		Sarcopenia			Muscle Mass	
	N	Diagnosis	Prevalence (%)	Diagnostic Criteria	Methods of Assessment	Threshold Values	Method of Assessment
Barone [51]	76	RA	21.0	EWGSOP 2010	MMI ^Ñ^, HGS	10.75 kg/m^2^ for men and 6.75 kg/m^2^ for women	BIA
Brance [47]	105	RA	19	EWGSOP 2019	SMI ^§^, HGS, 4MWS, sit-to-stand, TUG	7.0 kg/m^2^ for men and 5.5 kg/m^2^ for women	DXA
Cano-García [59]	76	RA	15.8	EWGSOP 2019	SMI ^§^, HGS, SPPB	7.0 kg/m^2^ for men and 5.5 kg/m^2^ for women	DXA
Chen [57]	238	RA	58.4	AWGS 2014, EWGSOP 2010	SMI ^§^	7.0 kg/m^2^ for men and 5.7 kg/m^2^ for women	BIA
Chu [63]	188	RA	63.8	AWGS 2014	SMI ^Ð^	7.0 kg/m^2^ for men and 5.7 kg/m^2^ for women	BIA
Dietzel [53]	289	RA	4.5/2.8	EWGSOP 2019/FNIH	SMI ^§^, SMI ^₩^, HGS, 6.45MWS, chair rise test	EWGSOP: 7.0 kg/m^2^ for men and 5.7 kg/m^2^ for women FNIH: 0.789 for men and 0.512 for women	DXA
Dobrovolskaya [65]	91	RA	27.5	EWGSOP 2019	SMI ^§^	6 kg/m^2^ for women	DXA
Ekici [66]	54	RA	31.5	EWGSOP 2019	SMMI ^œ^, HGS	6.76 kg/m^2^ for women and 10.76 kg/m^2^ for men	BIA
Fang [67]	64	RA	20.3	AWGS 2019	SMI ^§^, HGS, 6MWS	7.0 kg/m^2^ for men and 5.7 kg/m^2^ for women	BIA
Guzmán-Guzmán [54]	223	RA	86	EWGSOP 2010	SMI ^§^, HGS	7.26 kg/m^2^ for men and 5.45 kg/m^2^ for women	ΒΙA
Lian [48]	549	RA	61.7	AWGS 2014	SMI ^Ð^	based on the AWGS criteria	BIA
Lozada-Mellado [44]	165	RA	15.8	EWGSOP 2019	SMMI ^œ^, HGS	6.38 kg/m^2^ for women	ΒΙA
Mena-Vázquez [52]	94	RA	24.5	SMI	SMI ^§^	7.26 kg/m^2^ for men and 5.50 kg/m^2^ for women	DXA
Mochizuki [62]	240	RA	29.6	AWGS 2014	SMI ^§^, HGS, walking speed	7.0 kg/m^2^ for men and 5.7 kg/m^2^ for women	BIA
Mochizuki [68]	87	RA	10.3	AWGS 2014	SMI ^§^, HGS, walking speed	NR	BIA
Moschou [69]	80	RA	39	EWGSOP 2019	SMI ^§^, HGS, SPPB	5.45 kg/m^2^ for women	DXA
Nakayama [70]	2416	RA	14.1	SARC-F	N/A	N/A	N/A
Pardali [71]	79	RA	7.6	EWGSOP 2010	FFMI ^¤^, HGS	18 kg/m^2^ for men and 15 kg/m^2^ for women	Skinfold thickness
Qu [56]	480	RA	19.4	AWGS 2019	NR	NR	NR
Santos [72]	89	RA	4.5	FFMI	FFMI ^¤^	≤2 SD below the mean of a reference Caucasian sample	BIA
Tada [73]	100	RA	28	AWGS 2014	SMI ^§^, HGS, 3MWS	7.0 kg/m^2^ for men and 5.7 kg/m^2^ for women	BIA
Tekgoz [50]	100	RA	35 *	EWGSOP 2019	SMMI ^Ø^, HGS, 6MWS	9.2 kg/m^2^ for men and 7.4 kg/m^2^ for women	BIA
Tong [74]	474	RA	62.4 *	AWGS 2014	SMI ^§^	7.0 kg/m^2^ for men and 5.7 kg/m^2^ for women	BIA
Torii [58]	388	RA	37.1	EWGSOP 2010	SMI ^§^, HGS, 6MWS	7.0 kg/m^2^ for men and 5.7 kg/m^2^ for women	BIA
Tournadre [46]	74	RA	7.8	EWGSOP 2010	SMI ^§^, HGS, walking speed	7.26 kg/m^2^ for men and 5.45 kg/m^2^ for women	DXA
Tournadre [75]	21	RA	28.6	SMI	SMI ^§^	7.26 kg/m^2^ for men and 5.5 kg/m^2^ for women	DXA
Valencia-Muntalà [45]	67	RA	43.3/16.4/62.7	EWGSOP 2010/EWGSOP 2019/SARC-F	SMI ^§^, HGS, 6MWS	5.67 kg/m^2^ for women	DXA
Vlietstra [76]	82	RA	17.1	FNIH	SMI ^₩^, HGS, 40MWS, sit-to-stand, TUG	0.789 for men and 0.512 for women	DXA
Wiegmann [60]	238	RA	4.6/2.9	EWGSOP 2019/FNIH	SMI ^§^, SMI ^₩^, HGS, SPPB, 6.45MWS, TUG	SMI ^§^: 7.0 kg/m^2^ for men and 5.5 kg/m^2^ for women SMI ^₩^: 0.789 for men and 0.512 for women	DXA
Yamada [18]	100	RA	13.4	AWGS 2014	SMI ^§^, HGS, 3MWS	7.0 kg/m^2^ for men and 5.7 kg/m^2^ for women	BIA
Yun [49]	320	RA	2.2/6.6/11.9	AWGS 2019/EWGSOP 2010/SARC-F	SMI ^§^, HGS, 4MWS	AWGS: 7.0 kg/m^2^ for men and ≤5.7 kg/m^2^ for women EWGSOP: 8.87 kg/m^2^ for men and 6.42 kg/m^2^ for women	BIA
Zhang [61]	130	RA	43.1	AWGS 2014	SMI ^§^	7.0 kg/m^2^ for men and 5.7 kg/m^2^ for women	BIA

ALM: appendicular lean mass; N/A: not applicable; ASMI: appendicular skeletal muscle mass index; AWGS: Asian Working Group for Sarcopenia [24,26]; BIA: bioelectrical impedance analysis; BMI: body mass index; CAG: skinfold-corrected upper arm girth; CTG: skinfold-corrected thigh girth; CCG: skinfold-corrected calf girth; DXA: dual-energy X-ray absorptiometry; EWGSOP: European Working Group on Sarcopenia in Older People [3,4]; FFM: fat-free mass; FFMI: fat-free mass index; FNHI: Foundation for the National Institutes of Health [25]; HGS: handgrip strength; Ht: height; Ishii: estimate the probability of sarcopenia including three variables: age, grip strength, and calf circumference [77]; kg: kilogram; m: meter; MMI: muscle mass index; MWS: meter walking speed; NR: not reported; PsA: psoriatic arthritis; RA: rheumatoid arthritis; SARC-F: strength, assistance with walking, rise from a chair, climb stairs, and falls; SD: standard deviation; SMI: skeletal muscle index; SMMI: skeletal muscle mass index; SPPB: short physical performance battery; TUG: timed up-and-go. * increased risk of sarcopenia or pre-sarcopenia; ^Ñ^ MMI: total muscle mass/height^2^; ^§^ SMI: the sum of upper and lower limb muscle mass (ALM) divided by squared height (kg/m^2^); ^₩^ SMI: the sum of upper and lower limb muscle mass (ALM) divided by BMI (kg/[kg/m^2^]); ^Ð^ SMI: skeletal muscle mass/height^2^ (g/m^2^); ^œ^ SMMI: SMM/height^2^, where SMM = [(height^2^/R × 0.401) + (gender × 3.825) + (age × −0.071)] + 5.102, R is resistance, 0 = men and 1 = women; ^Ø^ SMMI = SMM/height^2^, where SMM = FFM × 0.566; ^¤^ FFMI: FFM divided by the square of the height (kg/m^2^).

### 5.2. Sarcopenia in Systemic Sclerosis (SSc)

SSc is an autoimmune condition with various clinical manifestations involving the skin, internal organs, musculoskeletal system, systemic inflammation, and gastrointestinal complications [78]. Heart, lung, and joint involvement, along with digital ulcers, hinder physical capacity and performance, ultimately leading to muscle atrophy [79].

According to a recent meta-analysis [80], sarcopenia affects 22% of patients with SSc and is associated with poorer quality of life and greater CRP levels. Depending on the diagnostic approaches employed in the primary studies, sarcopenia in SSc varied between 10.7 [81] and 52.9% [82] (Table 4). The findings of the studies associate sarcopenia with reduced HGS [82,83], decreased muscle mass [81,82], and impaired physical performance [83]. Patients with SSc and comorbid sarcopenia tend to exhibit reduced capillary density and peripheral blood flow, indicating compromised muscle health and function [84], alongside more severe lung and skin involvement [85]. Notably, most patients diagnosed with sarcopenia have diffuse cutaneous SSc and elevated CRP concentrations, suggesting a potential connection between sarcopenia and increased systemic inflammation [86]. Furthermore, in SSc, sarcopenia was associated with lower BMI [83] and malnutrition [82,83].

Hax and associates [87] suggested utilizing the SARC-F tool, adjusted for age and body mass (SARC-F + EBM), as a practical means for assessing sarcopenia in patients with SSc. Compared to SARC-F alone, which can lead to false-negative results due to SSc symptomatology, this adapted tool showed superior performance and higher sensitivity, while being feasible and easy to implement. Recent research [9,88] using computed tomography (CT) revealed that, in SSc, myopenia is associated with BMI, whereas another manifestation, namely myosteatosis, appears to be more prevalent and strongly associated with clinical features, including lung involvement and esophageal dilatation, indicating the importance of low muscle quality in scleroderma outcomes.

**Table 4:** Studies assessing the prevalence of sarcopenia in patients with SSc.

First Author	Sample		Sarcopenia			Muscle Mass	
	N	Diagnosis	Prevalence (%)	Diagnostic Criteria	Methods of Assessment	Threshold Values	Method of Assessment
Ajdynan [89]	43	SSc	33.3	EWGSOP 2019	SMI ^§^, HGS, sit-to-stand	NR	DXA
Caimmi [85]	140	SSc	20.7	SMI	SMI ^§^	7.26 kg/m^2^ for men and 5.50 kg/m^2^ for women	DXA
Corallo [82]	62	SSc	42	EWGSOP 2010	SMI ^§^, HGS	7.26 kg/m^2^ for men and 5.50 kg/m^2^ for women	DXA
Hax [87]	94	SSc	15.9/ 22.3/ 21.3/ 21.3/36.2	EWGSOP 2019/ SARC-F/ SARC-CalF/ SARC-F + EBM/Ishii	SMI ^§^, HGS, 4MWS, SPPB	7.0 kg/m^2^ for men and 5.50 kg/m^2^ for women	DXA
Paolino [84]	43	SSc	23.3	EWGSOP 2010	SMI ^§^	7.26 kg/m^2^ for men and 5.50 kg/m^2^ for women	DXA
Pardali [71]	17	SSc	52.9	EWGSOP 2010	FFMI ^¤^, HGS	18 kg/m^2^ for men and 15 kg/m^2^ for women	Skinfold thickness
Rincón [90]	27	SSc	33.3	EWGSOP 2010	SMI ^§^, HGS, 4MWS	7.26 kg/m^2^ for men and 5.50 kg/m^2^ for women	DXA
Sangaroon [86]	180	SSc	22.8	AWGS 2019	SMI ^§^, FFMI ^¤^, HGS, 6MWS	7.0 kg/m^2^ for men and 5.4 kg/m^2^ for women	DXA
Sari [81]	93	SSc	10.7	EWGSOP 2010	ASMI ^ 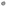 ^, HGS	7.26 kg/m^2^ for men and 5.50 kg/m^2^ for women	BIA
Siegert [83]	129	SSc	22.5	EWGSOP 2010	SMI ^§^, HGS	7.26 kg/m^2^ for men and 5.50 kg/m^2^ for women	BIA

ALM: appendicular lean mass; ASMI: appendicular skeletal muscle mass index; AWGS: Asian Working Group for Sarcopenia [24,26]; BIA: bioelectrical impedance analysis; BW: body weight; DXA: dual-energy X-ray absorptiometry; EWGSOP: European Working Group on Sarcopenia in Older People [3,4]; FFM: fat-free mass; FFMI: fat-free mass index; HGS: handgrip strength; Ishii: estimate the probability of sarcopenia including three variables: age, grip strength, and calf circumference [77]; kg: kilogram; m: meter; MWS: meter walking speed; NR: not reported; SARC-F: strength, assistance with walking, rise from a chair, climb stairs, and falls; SARC-CalFL: SARC-Calf combining calf circumference; SARC-F + EBM: SARC-F adding age and body mass; SMI: skeletal muscle index; SPPB: short physical performance battery; SSc: systemic sclerosis; ^
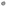
^ ASMΙ: ALM/height^2^ where ALM = −4.211 + (0.267 × height^2^/resistance) + (0.095 × BW) + (1.909 × sex [men = 1, women = 0]) + (−0.012 × age) + (0.058 × reactance); ^§^ SMI: the sum of upper and lower limb muscle mass (ALM) divided by squared height (kg/m^2^); ^¤^ FFMI: FFM divided by the square of the height (kg/m^2^).

### 5.3. Sarcopenia in Spondyloarthritides (SpAs)

SpAs consists of a group of inflammatory RDs that primarily affect the spine and sacroiliac joints. Aside from that, they can also involve peripheral joints, entheses, and extra-articular organs like the skin, eyes, and gastrointestinal tract [91]. Individuals with SpAs demonstrate reduced physical performance [92], along with decreased muscle mass [92,93] and muscle strength [92,93,94,95]. A higher prevalence of sarcopenia in patients with SpA was observed using the AWGS 2019 criteria (Table 5) and this correlated with greater Bath Ankylosing Spondylitis Functional Index scores, indicating mobility limitations, as well as with older age and lower BMI [93].

Ankylosing spondylitis (AS) is an inflammatory condition classified as a type of seronegative SpA [96]. It is associated with impaired physical performance and muscle strength [97]. In people with AS, sarcopenia was associated with reduced HGS [51,98], muscle mass [51,98], and diminished muscle capability [98]. Patients with sarcopenia were found to exhibit elevated CRP concentrations and greater mobility issues [51]. In men, sarcopenia was related to lower BMI and the presence of osteoporosis [98]. Pre-sarcopenia was estimated to be apparent in 50.4% of this population and was linked to lower disease activity (Bath AS disease activity index) [98], suggesting that greater disease activity might also propel muscle wasting.

On the other hand, psoriatic arthritis (PsA) is a condition within the spectrum of SpA, affecting approximately 0.11% of the global population [99]. The prevalence of sarcopenia in PsA ranges between 5.1% [100] and 62% [101] (Table 5), with performed assessments being based on both muscle mass [46,100,102] and function [46,100,102]. However, the reported prevalence rates of 62% [101] and 43.1% [102] are based solely on skeletal muscle mass measurements, without incorporating functional assessments. When considering studies that apply more comprehensive diagnostic criteria, the prevalence narrows down considerably, ranging between 5.1% [100] and 14.3% [71]. Sarcopenia in PsA has been linked to osteoporosis [100,102] and lower BMI in women [100]. Recent research has shown that approximately 12% of patients with PsA have osteoporosis [103]. In postmenopausal women with PsA, osteoporosis was found to be twice as prevalent among those with sarcopenia compared to those without [102].

**Table 5:** Studies assessing the prevalence of sarcopenia in patients with SpA.

First Author	Sample		Sarcopenia			Muscle Mass	
	N	Diagnoses	Prevalence (%)	Diagnostic Criteria	Methods of Assessment	Threshold Values	Method of Assessment
Aguiar [101]	36/ 24	AS/ PsA	62	MMI	MMI ª	Grade I: 8.51 < MMI < 10.75 for men and 5.76 < MMI < 6.75 for women; Grade II: MMI < 8.51 for men and <5.76 for women	Skinfold thickness
El Maghraoui [98]	67	AS	34.3/50.4 *	EWGSOP 2010	SMI ^§^, HGS, TUG	7.25 kg/m^2^ for men	DXA
Kanjanavaikoon [93]	104	SpA	22.1	AWGS 2019, SARC-F, SARC-CalF	SMI ^§^, calf circumference, 6MWS, sit-to-stand	7.0 kg/m^2^ for men 5.4 kg/m^2^ for women	DXA
Krajewska-Wlodarczyk [102]	51	PsA	13.7 (SMI ^§^) 43.1 (SMI ^š^)	EWGSOP 2010	SMI ^§^, SMI ^š^, TUG	SMI ^§^: 5.4 kg/m^2^ for women SMI ^š^: 27.6% for women	BIA
Merle [92]	103	SpA	5	EWGSOP 2019	SMI ^§^, HGS, 4MWS	7.0 kg/m^2^ for men and 5.5 kg/m^2^ for women	DXA
Pardali [71]	21	PsA	14.3	EWGSOP 2010	FFMI ^¤^, HGS	18 kg/m^2^ for men and 15 kg/m^2^ for women	Skinfold thickness
Pardali [71]	17	AS	11.8	EWGSOP 2010	FFMI ^¤^, HGS	18 kg/m^2^ for men and 15 kg/m^2^ for women	Skinfold thickness
Takami [100]	156	PsA	5.1/16.7 *	AWGS 2019	SMI ^§^, HGS	7.0 kg/m^2^ for men and 5.4 kg/m^2^ for women	DXA
Tournadre [46]	11	PsA	9.1	EWGSOP 2010	SMI ^§^, HGS, walking speed	7.26 kg/m^2^ for men and 5.45 kg/m^2^ for women	DXA
Tournadre [46]	63	SpA	1.7	EWGSOP 2010	SMI ^§^, HGS, walking speed	7.26 kg/m^2^ for men and 5.45 kg/m^2^ for women	DXA

AS: ankylosing spondylitis; AWGS: Asian Working Group for Sarcopenia [24,26]; BIA: bioelectrical impedance analysis; CAG: skinfold-corrected upper arm girth; CTG: skinfold-corrected thigh girth; CCG: skinfold-corrected calf girth; DXA: dual-energy X-ray absorptiometry; EWGSOP: European Working Group on Sarcopenia in Older People [3,4]; FFMI: fat-free mass index; HGS: handgrip strength; Ht: height; kg: kilogram; m: meter; MMI: muscle mass index; MWS: meter walking speed; SARC-F: strength, assistance with walking, rising from a chair, climb stairs, and falls; SARC-CalFL: SARC-Calf combining calf circumference; PsA: psoriatic arthritis; SMI: skeletal muscle index; SpAs: spondyloarthritides; TUG: timed up-and-go. * increased risk of sarcopenia or pre-sarcopenia; ^§^ SMI: the sum of upper and lower limb muscle mass (ALM) divided by squared height (kg/m^2^); ^š^ SMI: the ratio of the total skeletal muscle mass to the mass of the body expressed as a percentage; ^¤^ FFMI: FFM divided by the square of the height (kg/m^2^); ª MMI: muscle mass/height^2^ where muscle mass = Ht × (0.00744 × CAG^2^ + 0.00088 × CTG^2^ + 0.00441 × CCG) + 2.4 × sex − 0.048 × age + race + 7.8 where sex = 0 for female or 1 for male; age is in years; race = −2.0 for Asian, 1.1 for African American, and 0 for white and Hispanic.

### 5.4. Sarcopenia in Systemic Lupus Erythematosus (SLE)

SLE is a complex autoimmune disorder that affects multiple systems and internal organs [42]. Regarding the musculoskeletal system, individuals exhibit decreased muscle mass and strength and reduced physical function [104]. To our knowledge, four studies have investigated the prevalence of sarcopenia among patients with SLE, with the prevalence ranging between 10.9% [72] and 25% [71] (Table 6). The lowest prevalence was identified in a study that employed fat-free mass index (FFMI) as the sole diagnostic criterion [72]. Two studies using the EWGSOP 2019 criteria reported intermediate prevalence rates, reflecting a more conservative diagnostic approach [105,106]. In contrast, the highest prevalence was observed in a study applying the EWGSOP 2010 criteria and using skinfolds as the method of assessment of muscle mass [71]. Additionally, a meta-analysis comprising 11 primary studies examined muscle strength in individuals with SLE and revealed that most patients demonstrated diminished muscle strength, particularly in the presence of deforming arthropathy [104]. Some patients with SLE also develop lupus myositis, inflammation of muscle mass, leading to muscle wasting and weakness, propelling sarcopenia. Muscle mass deterioration and frailty have also been linked to reduced health-related quality of life, as well as increased levels of pain and fatigue [107]. These associations highlight the broader impact of musculoskeletal decline on both the physical and psychosocial well-being of affected individuals.

### 5.5. Sarcopenia in Juvenile Idiopathic Arthritis (JIA)

JIA refers to a group of arthritis subtypes with onset before the age of 16 [108], characterized by chronic joint inflammation and symptoms linked to systemic inflammatory processes [109]. Patients with JIA often present lower physical activity, malnutrition, lower BMI, and are undernourished [110]. Research on the prevalence of sarcopenia in JIA is presented in Table 7. Furthermore, sarcopenia in JIA has been strongly related to reduced total lean mass (which correlates with lower HGS), vitamin D concentration, disease activity as indicated by elevated ESR concentrations, and overall poor health [111]. Early-onset sarcopenia significantly impairs muscle development and poses lasting challenges for muscle health and functionality [112], indicating that management of the condition is crucial for improved patient outcomes.

### 5.6. Sarcopenia in Primary Sjögren’s Syndrome (pSS)

pSS is a systemic autoimmune inflammatory syndrome, mostly prevalent in women [113]. Its manifestations involve joint pain, fatigue, and dryness of the eyes and the mouth [113]. In pSS, both sarcopenia and pre-sarcopenia (Table 8) have been associated with greater pain sensation [114,115,116], fatigue [114], and dryness scores [114], alongside higher disease activity indexes [117]. In women, sarcopenia was associated with a higher risk of malnutrition and decreased muscle strength [117]. On the other hand, individuals with pre-sarcopenia exhibited limited physical performance and lower quality of life [98], suggesting that even before the development of the condition, health and quality of life were greatly diminished.

### 5.7. Sarcopenia in Myositis

Myositis refers to a group of rare diseases characterized by chronic inflammatory myopathy and progressive weakness of the skeletal muscles, which may also involve several internal organs including the lungs, heart, and esophagus [119]. Sarcopenia has been shown to affect more than 20% of the patients with myositis, with inactive or low disease activity, representing a subgroup with severe muscle involvement, significant disability, and higher treatment needs [120,121]. Research on sarcopenia in patients with myositis is presented in Table 9. Patients with myositis and sarcopenia exhibit greater muscle weakness, reduced physical performance, and increased damage scores, with impaired physical performance and higher HAQ scores. They more frequently experience myocarditis and require aggressive therapies such as intravenous immunoglobulins, plasmapheresis, and Janus kinase inhibitors [121]. Muscle mass, assessed by dual-energy X-ray absorptiometry (DXA), correlates with muscle strength and function, indicating that assessment of muscle mass can offer a quantitative tool to evaluate disease severity and disability in myositis [120,121,122]. Research using high-frequency ultrasound and shear wave elastography has shown that, in polymyositis and dermatomyositis, muscle properties are compromised as a result of disrupted proteostasis, with distinct muscle thickness and echo intensity that could also be used for the differential diagnosis between the two [123]. Nonetheless, the process of aging also contributes to inclusion body myositis (IBM) [124], further accelerating muscle atrophy. For this, Yamashita suggested that late-onset primary muscle diseases, such as inclusion body myositis, share common pathogenic mechanisms with sarcopenia, a fact that may often complicate the timely diagnostic process [125].

### 5.8. Sarcopenia in Fibromyalgia Syndrome (FMS)

Fibromyalgia syndrome (FMS) is a condition of unknown pathophysiology, characterized by chronic widespread pain sensation, neuroinflammation [126], stress and fatigue [127], memory loss, and sleep disturbances [128,129]. In patients with FMS (Table 10), pre-sarcopenia has been associated with impaired physical performance, as evidenced by deficits in the sit-to-stand and timed up-and-go (TUG) tests [130]. Additionally, those with greater pain severity and symptom burden, as assessed by the Fibromyalgia Impact Questionnaire, appeared to have lower HGS and reduced walking speed [131]. Moreover, neuromuscular pain associated with FMS may further contribute to disability, heightened fall risk, and increased susceptibility to injuries [132]. Similarly, in a study involving 45 individuals with FMS, participants demonstrated reduced HGS, poorer physical performance scores, and elevated scores on the SARC-F questionnaire [133].

Proteomics research has revealed that several proteins are actually dysregulated in FMS due to the excessive oxidative stress response [128], and this might further enhance muscle degradation and wasting, feeding sarcopenia.

### 5.9. Sarcopenia in Vasculitides

Vasculitides consist of a heterogeneous group of pathologies characterized by vessel inflammation [134]. The assessment of sarcopenia in patients with vasculitis remains insufficiently explored (Table 11). One study reported that while these patients exhibited reduced muscle strength, they did not demonstrate a decline in muscle mass, resulting in no cases meeting the criteria for sarcopenia [135]. Conversely, another study identified sarcopenia in 15.6% of patients [71]. While both studies used the EWGSOP 2010 criteria, they both used different methods of assessment regarding both sarcopenia and muscle mass. Nevertheless, low muscle strength was significantly associated with factors such as advanced age, disease severity, osteoporosis, markers of inflammation, malnutrition, and serious adverse events over a median follow-up of 42 months [135].

Research also indicates that myopathy may also be a common manifestation of vasculitis, further propelling sarcopenia. A recent systematic review [136] revealed that muscle involvement seldom occurs in large-vessel vasculitis, while it is more evident in Kawasaki or Behçet’s disease, with histological findings showing necrotizing vasculitis of the perimysium vessels. Vasculitic myopathy is also an issue of concern, with myopathy being the initial manifestation of vasculitis in approximately 80% of the patients, and weakness being symmetric in most of the cases [137]. This indicates that patients with vasculitides are particularly prone to sarcopenia when muscle involvement is apparent.

### 5.10. Sarcopenia in Other RDs and Mixed Patient Samples

Several studies have assessed sarcopenia in mixed RD patient samples (Table 12). A recent study revealed that sarcopenia was prevalent in 15.9% of patients with RDs [71]. Key contributors to sarcopenia included underweight status, a diagnosis of SSc, and patient-reported loss of appetite [71]. Furthermore, the risk of sarcopenia using the SARC-F was estimated at 23.9%, while the Mini Sarcopenia Risk Assessment (MSRA) was found to overestimate the risk of this condition [71]. Among hospitalized patients, one-month hospitalization with high-dose GC therapy was associated with sarcopenia progression, with early weight loss during the first week serving as a potential warning sign for muscle volume loss [138].

### 5.11. Sarcopenic Obesity

Sarcopenia combined with excessive fat accumulation (BMI > 30 kg/m^2^) results in a distinct condition known as sarcopenic obesity [140]. Excessively expanded white adipose tissues secrete pro-inflammatory cytokines, establishing a condition of low-grade inflammation [141]. Research on sarcopenic obesity in patients with RDs remains limited (Table 13), focusing mainly on the diagnoses with a greater obesity prevalence, like RA, where myopenia overlapping overfat consists of an important extra-articular manifestation in RA, particularly in patients on GC therapy [10]. On average, studies have identified sarcopenic obesity in 4.7% [89] to 44% [54] of patients with RDs. A recent cohort reported only one patient with sarcopenic obesity out of 220 in total, among a mixed RD sample [71]. In RA, sarcopenic obesity has been associated with greater HAQ and lower short physical performance battery (SPPB) [142], greater disease activity and morning stiffness, longer disease duration, rheumatoid factor (RF), anti-CCP (Cyclic Citrullinated Peptide) positiveness, and HAQ [54,65]. Patients with pre-sarcopenic obesity have been shown to exhibit reduced lean mass and to be more prone to malnutrition [59]. Both age and disease duration were positively correlated with the presence of sarcopenic obesity [59].

## 6. Sarcopenia and Pharmacological Treatments in RDs

Biologic disease-modifying anti-rheumatic drugs (bDMARDs) and, more recently, targeted synthetic DMARDs (tsDMARDs) and conventional synthetic DMARDs (csDMARDs) have changed the scene, advancing the management of RDs [143]. GCs, on the other hand, contribute to sarcopenia by promoting muscle wasting and protein degradation through various mechanisms [17,18].

### 6.1. The Class of DMARDs

Hasegawa et al. [144] reported that initiation of bDMARD therapy in RA patients with sarcopenia was associated with improvements in appendicular lean mass (ALM), skeletal muscle index (SMI), and a reduced prevalence of sarcopenia. However, the study did not differentiate between specific bDMARD agents, limiting conclusions about which therapeutic mechanisms were most effective. Similarly, findings by Torii et al. [58] identified a negative association between bDMARD use and sarcopenia, reinforcing the idea that they might offer muscle-protective benefits.

In contrast, a meta-analysis indicated that DMARDs had no significant impact on skeletal muscle mass in patients with RA [145]. A salient finding of this study was the demonstration that anti-IL-6 therapy exhibited a higher propensity for promoting an increase in lean body mass. Notably, one year of treatment with the IL-6 receptor inhibitor tocilizumab was associated with improvements in ALM, SMI, and fat-free mass index (FFMI), although it also led to increased subcutaneous fat accumulation [75]. The dual role of IL-6 in muscle metabolism may help explain these findings. In the context of chronic systemic inflammation, IL-6 primarily acts through its soluble receptor (sIL-6R), engaging the trans-signaling pathway. This mechanism is linked to pro-inflammatory and catabolic processes, particularly in joint and skeletal muscle tissues [146]. Therapeutic blockade of IL-6 signaling—especially the trans-signaling axis—may therefore mitigate inflammation-driven muscle degradation and serve as a targeted approach to address sarcopenia in RA. However, IL-6 is not exclusively pro-inflammatory; when released by skeletal muscle during exercise, it acts as a myokine which exerts anti-inflammatory and metabolic effects via the classical pathway [147].

A meta-analysis by Tekaya et al. [147] concluded that bDMARDs did not affect total muscle mass in individuals with RA or SpA. Similar results were also noted in another meta-analysis, which also failed to establish a clear association between b/tsDMARD use and sarcopenia [148]. In contrast, csDMARDs exhibited an inverse correlation with this condition [148]. However, the authors acknowledged that small sample sizes and lack of robust studies limited their conclusions [148].

Several studies have also reported no differences in current bDMARD use between patients with RA and sarcopenia and those without [62,68,69]. Furthermore, the utilization of tsDMARDs [62,68], methotrexate [44,62,68,69], hydroxychloroquine [69], leflunomide [44], sulfasalazine [44], or DMARDs as a general class [63] did not differ between these groups, further complicating attempts to establish a consistent pattern. Preclinical studies using animal models showed that the inhibition of the IL-6/JAK/STAT (signal transducer and activator of transcription) pathway has been associated with increased muscle mass in rabbit models of RA, suggesting a possible anti-catabolic effect of JAK inhibitors [149]. Nevertheless, these findings have not been consistently replicated in human clinical trials, and evidence supporting similar outcomes in patients remains limited and inconclusive [145].

Brance et al. found no association between bDMARD use and the presence of sarcopenia in patients with RA [47]. However, findings across the literature remain inconsistent. One study reported an association between TNF inhibitor use and reduced muscle mass [49], while another identified a positive association between bDMARD use and an increased risk of sarcopenia [70]. The role of TNF-α in muscle physiology may help explain some of this variability. Its effects appear to be concentration-dependent: at higher levels, TNF-α promotes cellular proliferation, whereas lower concentrations may support myogenic differentiation, particularly in the context of tissue repair, aging, or injury [32,150]. A prospective study examining the effects of TNF inhibitor therapy over 12 months observed increases in SMI, FFMI, and total lean mass, along with a concurrent rise in subcutaneous adipose tissue [151]. Nonetheless, whether these improvements are maintained over the long term remains uncertain, as the broader literature presents conflicting and inconclusive results.

### 6.2. Steroids and Muscle Wasting

GCs have been widely studied for their association with sarcopenia and muscle wasting. Numerous studies have reported a positive correlation between the use of GCs and the presence of sarcopenia and/or loss of muscle mass [18,47,70,148,152], implicating these agents as contributing factors in the progression of musculoskeletal decline [153]. Several studies have reported higher rates of current GC use among individuals with sarcopenia compared with those without [48,63,142], while others failed to report notable differences. Beyond muscle effects, GCs have also been associated with adverse changes in body composition, including early and accelerated reductions in bone mineral density and increased fat mass, particularly in patients with SLE [154]. In the CHIKARA study (Correlation research of sarcopenia, skeletal muscle, and disease activity in rheumatoid arthritis), the average GC dose over one year was linked to sarcopenia; however, disease activity showed no difference [18]. Moreover, Baker and associates identified a link between GC use and sarcopenic obesity, lower lean mass, higher HAQ, and lower physical performance as measured by the SPPB [142].

In a study of 129 individuals with SSc, the use of immunosuppressants varied between those with and without sarcopenia. Patients with sarcopenia were using an average of two immunosuppressants, compared to one in those belonging to the non-sarcopenic group [83]. However, no differences were observed between steroid and non-steroid immunosuppressants [83].

## 7. Lifestyle Approaches to Managing Sarcopenia

### 7.1. Nutritional Interventions

Evidence underlines the importance of nutrition in muscle synthesis, degradation, function, and strength [155]. Individuals with sarcopenia often present deficiencies in proteins, lipids, and essential micronutrients, including potassium, magnesium, phosphorus, iron, and vitamin K [156]. The importance of polyunsaturated fatty acids, antioxidants, and minerals in supporting muscle synthesis is also well recognized [157,158]. Furthermore, dietary patterns appear to influence muscle turnover, with diets rich in fruits and vegetables offering essential micronutrients and phytochemicals crucial for muscle function [157,158]. Notably, adherence to the Mediterranean diet has been associated with improved physical function [159] and enhanced walking performance [160].

Protein supplies the essential building blocks for muscle growth and helps maintain the balance between anabolism and catabolism. Dairy protein in particular has been suggested to improve ALM [161]. Leucine supplementation or leucine-enriched proteins have been shown to ameliorate symptoms of sarcopenia in older adults [162]. Administration of leucine and vitamin D, along with whey protein, improved ALM and the sit-and-stand test compared to a control group of older adults with sarcopenia [163]. Similarly, diets enriched with leucine and vitamin D improved muscle mass and lower limb function in individuals with sarcopenia [163]. Furthermore, combining whey protein, vitamin D, and vitamin E has enhanced muscle mass and quality of life [164]. A meta-analysis examining supplementation with whey protein, leucine, and vitamin D in patients with sarcopenia revealed that it can enhance ALM [165]. Additionally, when combined with a physical exercise program, it can further improve muscle strength and function [165]. Creatinine supplementation also contributes to improved body composition in RA, by increasing ALM [166].

Omega-3 fatty acids help prevent low-grade chronic inflammation and support muscle function by enhancing insulin sensitivity and exerting anti-inflammatory effects [167]. This is evidenced by the reduction in inflammatory markers such as IL-6, IL-1β, and TNF [168]. However, the underlying mechanisms of these effects remain unclear [167]. Individuals with sarcopenia tend to have lower n-3 fatty acid dietary intake compared to healthy controls [169]. An increase in muscle mass gain and an improvement in walking speed has been observed in doses >2 g n-3 fatty acids/day for a duration of 6 months in older individuals [170]. Nevertheless, a recent meta-analysis revealed that supplementation with n-3 fatty acids benefits whole-body protein synthesis rates but not muscle protein synthesis [171]. Intriguing findings also support lower-body strength improvement and not lean mass development [172]. A novel food combining leucine, n-3 fatty acids, and probiotic *Lactobacillus paracasei* PS23 consumed for two months was also shown to enhance ALM, muscle strength, and physical activity in elderly individuals with sarcopenia [173].

β-hydroxy-β-methylbutyrate (HMB) has also been proposed as a potential intervention against frailty in older adults with sarcopenia, improving lean muscle mass [174]. Supplementation with 1.5 g HMB b.i.d. for 12 weeks significantly augmented muscle strength and quality, physical performance, and reduced inflammation biomarkers [175]. A meta-analysis assessing HMB supplementation in individuals with sarcopenia revealed improvements in muscle strength [176].

### 7.2. Exercise as an Intervention

Physical activity protects against muscle frailty and the development of sarcopenia [177]. Several studies have explored the role of movement practices in enhancing muscle mass and addressing sarcopenia. A systematic review revealed that exercise interventions contribute to increased muscle mass and physical performance, while addressing nutrition and the workout practice provides additional advantages [178]. It has also been linked to better body performance and a reduction in fat mass [179].

Resistance training or a combination of moderate-to-high aerobic and resistant exercise performed at 50% or greater intensity is suggested for improving muscle strength in patients with RA, with no adverse effects in implementing such interventions [180,181,182,183]. However, disease duration may influence the impact of the intervention [184]. A recent study assessed a program incorporating Tai Chi-based physical activity, a supportive psychosocial approach, and interactive counseling in postmenopausal women with sarcopenia and RA, observed improved health-related quality of life and the disease activity score, along with progression in physical performance and lean mass advances [185]. Non-weight-bearing activities may offer therapeutic benefits for this population. A 16-week program consisting of water-based aerobic exercises in women with RA resulted in significant improvements in disease activity and functional capacity; however, these were not accompanied by an increase in knee muscle strength [186].

**Figure 3:**
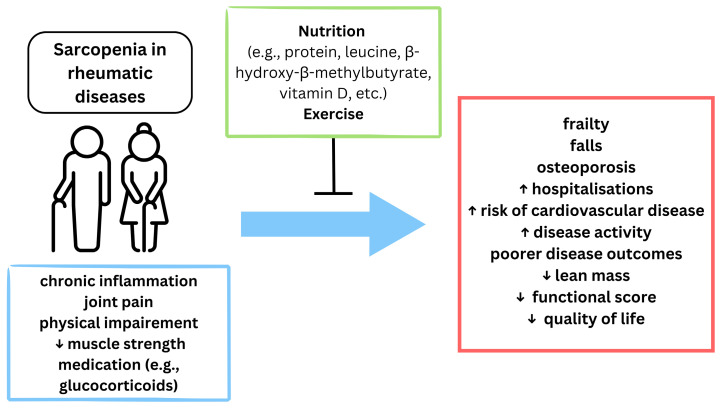
The impact and management of sarcopenia in rheumatic diseases. ↑ increased; ↓ reduced.

Figure 3 presents the relationship between sarcopenia in RDs and its consequences, highlighting the role of nutrition and exercise in mitigating progression and adverse outcomes.

## 8. Conclusions

Sarcopenia is prevalent among patients with RDs and is associated with significant adverse events and poor health outcomes. It results not only in a decrease in muscle mass, which in turn contributes to reduced mobility and a worsening prognosis of the disease, but also in complications such as cachexia and osteoporosis, amongst others. In addition to managing their RD symptoms, this clinical population often faces the additional challenge of pharmacological therapies that may further impair muscle health. Moreover, chronic pain may hinder their ability to participate in targeted interventions, worsening their condition.

## 9. Future Directions and Limitations

Sarcopenia should be recognized as a clinical condition, given its impact on overall health when it occurs primarily and its influence on disease outcomes when it develops secondarily. Healthcare providers should be informed regarding this condition and trained in its diagnosis and management, while interdisciplinary collaboration is required. Despite its importance, routine screening for sarcopenia is not yet a standard practice in hospitals. Educating rheumatologists on how to screen and manage sarcopenia—including nutritional interventions, oral nutrition supplements, and exercise—will provide better support to patients, potentially improving their functional abilities and quality of life. Finally, adopting modern definitions of sarcopenia—beyond just muscle mass—that are easy to use, universally accepted, and low-cost would be beneficial for achieving more consistent and widely applicable results.

## Figures and Tables

**Table 1:** Diagnostic criteria and assessment methods used for sarcopenia evaluation in patients with rheumatic diseases.

Diagnostic Criteria	Muscle Mass Determination	Threshold Values		Method(s) of Assessment
		Men	Women	
EWGSOP 2010	SMMI ^œ^	-	6.42 kg/m^2^	BIA
	SMI ^§^	7.23–8.87 kg/m^2^	5.45–6.75 kg/m^2^	DXA, BIA
	FFMI ^¤^	18 kg/m^2^	15 kg/m^2^	Skinfold thickness, DXA
	MMI ^Ñ^	10.75 kg/m^2^	6.75 kg/m^2^	BIA
	SMI ^š^	-	27.6%	BIA
	ASMI	7.26 kg/m^2^	5.5 kg/m^2^	BIA
EWGSOP 2019	SMI ^§^	7.0 kg/m^2^	5.5–5.7 kg/m^2^	DXA, BIA
	SMMI ^œ^	10.76 kg/m^2^	6.76 kg/m^2^	BIA
	SMMI ^Ø^	9.2 kg/m^2^	7.4 kg/m^2^	BIA
	SMI ^§^	6.0 kg/m^2^	5.5 kg/m^2^	DXA
AWGS 2014	SMI ^§^	7.0 kg/m^2^	5.7 kg/m^2^	BIA or DXA
AWGS 2019	SMI ^§^	7.0 kg/m^2^	5.4–5.7 kg/m^2^	BIA or DXA
FNIH	SMI ^₩^	0.789 kg/m^2^	0.512 kg/m^2^	DXA
SARC-F	N/A	N/A	N/A	N/A
SMI	SMI ^§^	7.26 kg/m^2^	5.5 kg/m^2^	DXA
MMI	MMI ª	Grade I: 8.51–10.75 kg/m^2^ Grade II: <8.51 kg/m^2^	Grade I: 5.76–6.75 kg/m^2^ Grade II: <5.76 kg/m^2^	Skinfold thickness
FFMI	FFMI ^¤^	≤2 SD below the mean of a reference Caucasian sample		BIA

ASMI: appendicular skeletal muscle index; AWGS: Asian Working Group for Sarcopenia; BIA: bioelectrical impedance analysis; DXA: dual-energy X-ray absorptiometry; EWGSOP: European Working Group on Sarcopenia in Older People; FFMI: fat-free mass index; FNIH: Foundation for the National Institutes of Health; kg: kilogram; m: meter; MMI: muscle mass index; N/A: not applicable; SARC-F: strength, assistance with walking, rise from a chair, climb stairs, falls questionnaire; SD: standard deviation; SMI: skeletal muscle index; SMMI: skeletal muscle mass index; SSMI: skeletal muscle mass index. ^Ñ^ MMI: total muscle mass/height^2^; ^§^ SMI: the sum of upper and lower limb muscle mass (ALM) divided by squared height (kg/m^2^); ^₩^ SMI: the sum of upper and lower limb muscle mass (ALM) divided by BMI (kg/[kg/m^2^]); ^š^ SMI: skeletal muscle mass/height^2^ (g/m^2^); ^œ^ SMMI: SMM/height^2^, where SMM = [(height^2^/R × 0.401) + (gender × 3.825) + (age × −0.071)] + 5.102, R is resistance, 0 = men and 1 = women; ^Ø^ SMMI: SMM/height^2^, where SMM = FFM × 0.566; ^¤^ FFMI: FFM divided by the square of the height (kg/m^2^); ª MMI: muscle mass/height^2^, with muscle mass = Ht × (0.00744 × CAG^2^ + 0.00088 × CTG^2^ + 0.00441 x CCG) + 2.4 × sex −0.048 × age + race + 7.8 where sex = 0 for female and 1 for male; age is in years; race = −2.0 for Asian, 1.1 for African American, and 0 for white and Hispanic.

**Table 2:** Inflammation biomarkers associated with sarcopenia.

Diagnostic Criteria	Biological Function	Association with Sarcopenia
IL-6	cytokine involved in inflammation and myokine signaling	suppresses IGF-1 and activates JAK/STAT pathway, increasing myolytic enzyme expression and muscle catabolism
TNF-α	pro-inflammatory cytokine	activation of NF-κB, muscle apoptosis, and breakdown via downregulation of myogenic and upregulation of myolytic enzymes
IL-1β	pro-inflammatory cytokine involved in immune signaling	promotes catabolic activity in muscle, contributing to muscle wasting and sarcopenia
IL-8	chemokine involved in inflammation and neutrophil recruitment	elevated levels linked to neutrophil dysregulation, muscle damage, and sarcopenia
CRP	acute-phase protein; marker of systemic inflammation	elevated levels associated with muscle degradation, sarcopenia, and frailty
ESR	non-specific marker of systemic inflammation	elevated levels linked to sarcopenia, reduced strength, and poor physical performance
Adiponectin-to-leptin ratio	anti- and pro-inflammatory adipokines	adiponectin inhibits NF-kB, while leptin stimulates the secretion of IL-6 and TNF-α
Neutrophil-to-lymphocyte ratio	indicator of immune system status and systemic inflammation	a greater ratio is negatively associated with fat-free mass

CRP: C-reactive protein; ESR: erythrocyte sedimentation rate; IGF-1: insulin-like growth factor 1; IL-1β: interleukin-1 beta; IL-6: interleukin-6; IL-8: interleukin-8; JAK: Janus kinase; NF-κB: nuclear factor-kappa B; NLR: neutrophil-to-lymphocyte ratio; STAT: signal transducer and activator of transcription; TNF-α: tumor-necrosis factor-alpha.

**Table 6:** Studies assessing the prevalence of sarcopenia in patients with SLE.

First Author	Sample		Sarcopenia			Muscle Mass	
	N	Diagnosis	Prevalence (%)	Diagnostic Criteria	Methods of Assessment	Threshold Values	Method of Assessment
Bilici [105]	82	SLE	12.9	EWGSOP 2019	SMI ^₩^, HGS	0.823 (kg/[kg/m^2^]) for women	BIA
Pardali [71]	28	SLE	25	EWGSOP 2010	FFMI ^¤^, HGS	18 kg/m^2^ for men and 15 kg/m^2^ for women	Skinfold thickness
Pena [106]	49	SLE	16.3	EWGSOP 2019	FFMI ^¤^, HGS, chair rise test	<15 kg/m^2^ for women	DXA
Santos [72]	92	SLE	10.9	FFMI	FFMI ^¤^	≤2 SD below the mean of a reference Caucasian sample	BIA

BIA: bioelectrical impedance analysis; BMI: body mass index; DXA: dual-energy X-ray absorptiometry; EWGSOP: European Working Group on Sarcopenia in Older People [3,4]; FFMI: fat-free mass index; HGS: handgrip strength; kg: kilogram; m: meter; SD: standard deviation; SMI: skeletal muscle index; SLE: systemic lupus erythematosus; ^₩^ SMI: the sum of upper and lower limb muscle mass (ALM) divided by BMI (kg/[kg/m^2^]). *^¤^* FFMI: FFM divided by the square of the height (kg/m^2^).

**Table 7:** Studies assessing the prevalence of sarcopenia in patients with JIA.

First Author	Sample		Sarcopenia			Muscle Mass	
	N	Diagnosis	Prevalence (%)	Diagnostic Criteria	Methods of Assessment	Threshold Values	Method of Assessment
Kulyk [111]	70	JIA	59	EWGSOP 2019	SMI ^§^, HGS, 4MWS	7.0 kg/m^2^ for men and 5.7 kg/m^2^ for women	DXA
Slitine [112]	34	JIA	20.6/11.7	EWGSOP 2019	SMI ^§^, HGS, SARC-F	EWGSOP: 7.0 kg/m^2^ for men and 5.5 kg/m^2^ for women and Alternative: SMI-2SD compared with age- and sex-matched reference values from a Danish cohort	DXA

DXA: dual-energy X-ray absorptiometry; EWGSOP: European Working Group on Sarcopenia in Older People [3,4]; HGS: handgrip strength; JIA: juvenile idiopathic arthritis; kg: kilogram; m: meter; MWS: meter walking speed; SARC-F: strength, assistance with walking, rising from a chair, climb stairs, and falls; SD: standard deviation; SMI: skeletal muscle index; ^§^ SMI: the sum of upper and lower limb muscle mass (ALM) divided by squared height (kg/m^2^).

**Table 8:** Studies assessing the prevalence of sarcopenia in patients with pSS.

First Author	Sample		Sarcopenia			Muscle Mass	
	N	Diagnosis	Prevalence (%)	Diagnostic Criteria	Methods of Assessment	Threshold Values	Method of Assessment
Alunno [118]	144	pSS	27	SARC-F	SARC-F	N/A	N/A
Colak [117]	44	pSS	25	EWGSOP 2019	SMMI ^Ø^, HGS, 6MWS	9.2 kg/m^2^ for men and 7.4 kg/m^2^ for women	BIA
Öztürk [116]	49	pSS	28.5	EWGSOP 2019	SMMI ^Ø^, HGS, 4MWS, sit-to-stand	9.2 kg/m^2^ for men and 7.4 kg/m^2^ for women	BIA

BIA: bioelectrical impedance analysis; EWGSOP: European Working Group on Sarcopenia in Older People [3,4]; FFM: fat-free mass; HGS: handgrip strength; kg: kilogram; m: meter; MWS: meter walking speed; N/A: not applicable; pSS: primary Sjögren’s syndrome; SARC-F: strength, assistance with walking, rising from a chair, climb stairs, and falls; SMM: skeletal muscle mass; SMMI: skeletal muscle mass index; ^Ø^ SMMI: SMM/height^2^, where SMM = FFM × 0.566.

**Table 9:** Studies assessing the prevalence of sarcopenia in patients with myositis.

First Author	Sample		Sarcopenia			Muscle Mass	
	N	Diagnosis	Prevalence (%)	Diagnostic Criteria	Methods of Assessment	Threshold Values	Method of Assessment
Giannini [122]	29	myositis	13.8	EWGSOP 2019	SMI ^§^, HGS, 6MWT, number of squats	NR	DXA
Giannini [121]	34	myositis	20.6	EWGSOP 2019	SMI ^§^, HGS, 6MWT, number of squats	NR	DXA
Giannini [120]	40	myositis	17.5	EWGSOP 2019	SMI ^§^, HGS, 6MWT	NR	DXA

6MWT: 6 min walk test; DXA: dual-energy X-ray absorptiometry; EWGSOP: European Working Group on Sarcopenia in Older People [3,4]; HGS: handgrip strength; kg: kilogram; m: meter; NR: not reported; SMI: skeletal muscle index; ^§^ SMI: the sum of upper and lower limb muscle mass (ALM) divided by squared height (kg/m^2^).

**Table 10:** Studies assessing the prevalence of sarcopenia in patients with FMS.

First Author	Sample		Sarcopenia			Muscle Mass	
	N	Diagnosis	Prevalence (%)	Diagnostic Criteria	Methods of Assessment	Threshold Values	Method of Assessment
Kapuczinski [133]	45	FMS	0	EWGSOP 2010	SMMI ^œ^, HGS, SPPB, SARC-F	6.42 kg/m^2^ for women	ΒΙA
Koca [131]	150	FMS	8.7	EWGSOP 2010	SMI ^§^, HGS, MWS	6.75 kg/m^2^ for women	BIA

BIA: bioelectrical impedance analysis; EWGSOP: European Working Group on Sarcopenia in Older People [3]; FMS: fibromyalgia syndrome; HGS: handgrip strength; kg: kilogram; m: meter; MWS: meter walking speed; SARC-F: strength, assistance with walking, rising from a chair, climb stairs, and falls; SMI: skeletal muscle index; SMM: skeletal muscle mass; SMMI: skeletal muscle mass index; SPPB: short physical performance battery; ^§^ SMI: the sum of upper and lower limb muscle mass (ALM) divided by squared height (kg/m^2^); ^œ^ SMMI: SMM/height^2^, where SMM = [(height^2^/ R × 0.401) + (gender × 3.825) + (age × − 0.071)] + 5.102, R is resistance, 0 = men and 1 = women.

**Table 11:** Studies assessing the prevalence of sarcopenia in patients with vasculitis.

First Author	Sample		Sarcopenia			Muscle Mass	
	N	Diagnosis	Prevalence (%)	Diagnostic Criteria	Methods of Assessment	Threshold Values	Method of Assessment
Henriquez [135]	120	vasculitis	0	EWGSOP 2010	SMI ^§^, HGS	7.23 kg/m^2^ for men and 5.67 kg/m^2^ for women	DXA
Pardali [71]	32	vasculitis	15.6	EWGSOP 2010	FFMI ^¤^, HGS	18 kg/m^2^ for men and 15 kg/m^2^ for women	Skinfold thickness

DXA: dual-energy X-ray absorptiometry; EWGSOP: European Working Group on Sarcopenia in Older People [3]; FFMI: fat-free mass index; HGS: handgrip strength; kg: kilogram; m: meter; SMI: skeletal mass index; ^¤^ FFMI: FFM divided by the square of the height (kg/m^2^); ^§^ SMI: the sum of upper and lower limb muscle mass (ALM) divided by squared height (kg/m^2^).

**Table 12:** Studies assessing the prevalence of sarcopenia in patients with mixed RD samples.

First Author	Sample		Sarcopenia			Muscle Mass	
	N	Diagnoses	Prevalence (%)	Diagnostic Criteria	Methods of Assessment	Threshold Values	Method of Assessment
Hanaoka [138]	49	RDs	83.7	AWGS 2019	SMI ^§^	7.0 kg/m^2^ for men and 5.4 kg/m^2^ for women	DXA
Pardali [71]	220	RDs	15.9	EWGSOP 2010	FFMI ^¤^, HGS	18 kg/m^2^ for men and 15 kg/m^2^ for women	Skinfold thickness
Ureña [139]	46	RDs	26	NR	NR	NR	DXA

AWGS: Asian Working Group for Sarcopenia [24,26]; BIA: bioelectrical impedance analysis; DXA: dual-energy X-ray absorptiometry; EWGSOP: European Working Group on Sarcopenia in Older People [3,4]; FFMI: fat-free mass index; HGS: handgrip strength; kg: kilogram; m: meter; NR: not reported; RD: rheumatic diseases; SARC-F: strength, assistance with walking, rising from a chair, climb stairs, and falls; SMI: skeletal muscle index; SMMI: skeletal muscle mass index; SPPB: short physical performance battery; ^§^ SMI: the sum of upper and lower limb muscle mass (ALM) divided by squared height (kg/m^2^); ^¤^ FFMI: FFM divided by the square of the height (kg/m^2^).

**Table 13:** Studies assessing the prevalence of sarcopenic obesity in patients with RDs.

First Author	Sample		Sarcopenic Obesity Prevalence (%)
	N	Diagnoses	
Ajdynan [89]	43	SSc	4.7
Baker [142]	444	RA	12.6
Dobrovolskaya [65]	91	RA	18.7
Guzmán-Guzmán [54]	223	RA	44
Mena-Vázquez [52]	94	RA	NR
Santos [72]	92/89	SLE/RA	6.5/5.6
Pardali [71]	220	Mixed RDs	0.05
Vlietstra [76]	82	RA	15.8

NR: not reported; RA: rheumatoid arthritis; RD: rheumatic disease; SLE: systemic lupus erythematosus.

## Abbreviations

The following abbreviations are used in this manuscript:

ALM	Appendicular lean mass
AS	Ankylosing spondylitis
AWSG	Asian Working Group for Sarcopenia
BIA	Bioelectrical impedance analysis
bDMARDs	Biologic disease-modifying anti-rheumatic drugs
BMI	Body mass index
CHIKARA	Correlation research of sarcopenia, skeletal muscle, and disease activity in rheumatoid arthritis
CRP	C-reactive protein
csDMARDs	Conventional synthetic disease-modifying anti-rheumatic drugs
CCP	Cyclic Citrullinated Peptide
CT	Computed tomography
DXA	Dual-energy X-ray absorptiometry
ESPEN	European Society for Clinical Nutrition and Metabolism
ESR	Erythrocyte sedimentation rate
EWGSOP	European Working Group on Sarcopenia in Older People
FFMI	Fat-free mass index
FMS	Fibromyalgia syndrome
FNIH	Foundation For the National Institutes of Health Sarcopenia Project
GC	Glucocorticoids
HGS	Handgrip strength
HMB	Β-hydroxy-β-methylbutyrate
IGF-1	Insulin-like growth factor 1
IL-1β	Interleukin-1β
IL-6	Interleukin-6
IL-8	Interleukin-8
JAK	Janus kinase
JIA	Juvenile idiopathic arthritis
MMI	Muscle mass index
MRI	Magnetic resonance imaging
MSRA	Mini sarcopenia risk assessment
NF-kB	Nuclear factor-kappa b
PsA	Psoriatic arthritis
pSS	Primary Sjögren’s syndrome
RA	Rheumatoid arthritis
RDs	Rheumatic diseases
SARC-F	Strength, Assistance with Walking, Rise from a Chair, Climb Stairs, and Falls
SARC-F + EBM	SARC-F plus elderly and BMI
sIL6R	Soluble form of IL6 receptor
SLE	Systemic lupus erythematosus
SMI	Skeletal mass index
SpA	Spondyloarthritides
SPPB	Short physical performance battery
SSc	Systemic sclerosis
TNF-α	Tumor-necrosis factor-α
tsDMARDs	Targeted synthetic disease-modifying anti-rheumatic drugs
TUG	Timed up-and-go
